# Vegetation-Associated Mercury Patterns and Biogeochemical Signals in a Tropical Urban Mining Region

**DOI:** 10.1021/acsomega.6c07837

**Published:** 2026-10-17

**Authors:** Jerusa Schneider, Sheyla dos Santos Brito, André Massanobu Ueno Kunifoshita, Ricardo Lopes Tortorela de Andrade, Anne-Hélène Fostier, James Lambert-Smith, Maria José Maluf de Mesquita

**Affiliations:** † Department of Geology and Natural Resources (DGRN), Institute of Geosciences, 28132 University of Campinas (UNICAMP), Rua Carlos Gomes, 250, Campinas, São Paulo CEP 13083-855, Brazil; ‡ Graduate Program in Environmental Sciences, Institute of Natural, Human and Social Sciences, 583431 Federal University of Mato Grosso (UFMT), Av. Alexandre Ferronato, 1200, Sinop, Mato Grosso CEP 78557-267, Brazil; § Department of Analytical Chemistry, Institute of Chemistry, University of Campinas (UNICAMP), P.O. Box 6154, Campinas, São Paulo CEP 13083-970, Brazil; ∥ School of Earth and Environmental Sciences, 2112 Cardiff University, Main Building, Park Place, Cardiff, Wales CF10 3AT, U.K.

## Abstract

To evaluate vegetation-associated mercury (Hg) loading in tropical regions affected by artisanal and small-scale gold mining (ASGM), we quantified total mercury (HgT) in canopy leaves and leaf litter of *Licania tomentosa* across an ASGM-impacted urban center, Peixoto de Azevedo (PEI), and two lower-exposure reference sites: Nova Santa Helena (ASH) and a native forest fragment (ANM). Canopy leaves reached up to 543 μg kg^–1^ near gold-buying shops, with probabilistic modeling identifying a high-concentration subpopulation extending to 607 μg kg^–1^. Leaf litter consistently exhibited higher HgT than paired canopy leaves (Ratio_C_ = 1.39–4.70; mean = 3.15), indicating relative enrichment during senescence. Spatial interpolation suggested sharply localized vegetation-associated Hg hotspots within ∼300 m of gold-trading units, superimposed on a heterogeneous urban baseline. The regional baseline was defined using ASH, where HgT concentrations ranged from 6.82 to 35.31 μg kg^–1^ after excluding a single transport-related outlier. These results demonstrate that gold-trading corridors are plausible localized contributors to elevated HgT in urban vegetation and may produce Hg-enriched biomass that can enter municipal waste streams. This framework provides a high-resolution, low-cost approach for Hg surveillance in tropical ASGM regions and offers chemically grounded insight into vegetation-associated Hg patterns and biogeochemical partitioning in urban environments.

## Graphical abstract

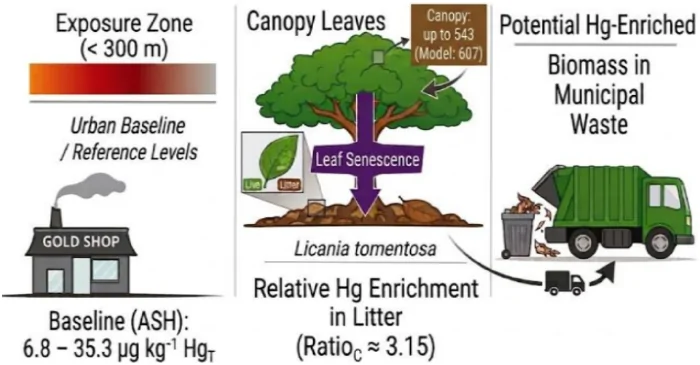

## 1 Introduction

Mercury (Hg) is a hazardous environmental contaminant with well-documented neurotoxic and bioaccumulative properties, posing persistent risks to ecosystems and human health due to its atmospheric mobility and long residence time.^1,2^ Artisanal and small-scale gold mining (ASGM) represents one of the largest global sources of anthropogenic Hg emissions, particularly in tropical regions, where amalgamation and secondary heating of Au–Hg alloys release substantial quantities of gaseous elemental mercury (Hg^0^) directly into the urban atmosphere.^2–4^ Previous biomonitoring studies conducted in the town of Alta Floresta (Brazil) using *Tillandsia usneoides* have demonstrated extreme Hg accumulation near gold shops, consistent with the spatial influence of ASGM -related activities.^3,5^ However, while epiphytes reflect short-term atmospheric concentration pulses, they lack connectivity to terrestrial accumulation pathways, limiting our understanding of how urban vegetation integrates both internal assimilation and externally deposited material.^3,5^ Although emissions are most concentrated at source, atmospheric transport and deposition can contaminate environments far beyond mining zones,^6,7^ providing important context for interpreting vegetation-associated total mercury (HgT) patterns in ASGM-impacted regions.

The biogeochemical cycle of Hg in terrestrial ecosystems is complex, with its fate mediated by atmospheric deposition and retention in vegetation and soils.^8^ Plant canopies play a critical role in intercepting atmospheric Hg, which is subsequently transferred to the soil via throughfall and leaf litter.^1,9^ Although Hg dynamics in forested environments are relatively well studied,^10–12^ current synthesis highlights the uniqueness of the Southern Hemisphere and the persistent data gaps in tropical regions, where shorter atmospheric lifetimes make Hg a predominantly hemispheric-scale pollutant.^12^ International literature has increasingly called for high-resolution data sets from tropical environments to reconcile global transport models; thus, understanding vegetation-associated Hg patterns and their relationship to canopy and litter compartments is important for improving assessments of terrestrial Hg sinks. Far less is known about Hg behavior in tropical urban landscapes impacted by ASGM, where high population density coincides with concentrated Hg emissions from gold-buying shops, informal amalgam burning, and unauthorized processing activities, creating conditions for acute and chronic exposure.^8,13^ Despite long-term monitoring in industrialized Brazilian cities showing that wet deposition is a significant pathway,^13^ spatially resolved data on vegetation-associated HgT in ASGM-impacted cities remain scarce, limiting the ability to identify areas of elevated HgT and to support informed environmental management strategies.^14–16^

Although *M. tomentosa* (syn. *Licania tomentosa*) has been previously used as a biomonitor in regional surveys across northern Mato Grosso,^17^ which studied only bulk leaf Hg concentrations across multiple municipalities and did not investigate spatial gradients, point-source influences, ASGM-related emissions, or leaf-litter dynamics. High-resolution urban-scale assessments capable of resolving localized deposition plumes near gold-trading infrastructure in the southern hemisphere therefore remain absent from the literature. Validating widespread, perennial canopy trees at the city scale provides a highly transferable, low-cost methodology for environmental surveillance in data-scarce urban centers across the global south.

Here we address this gap by mapping the spatial patterns of vegetation-associated HgT in two ASGM-intensive towns within the Alta Floresta Mineral Province in the Amazonas Craton,^18^ thereby investigating gradients consistent with atmospheric influence and vertical transfer between canopy leaves and litter. Using the widespread urban tree *Licania tomentosa*, a Brazilian native, as a biomonitor, we quantified HgT in both green canopy leaves and senescent leaf litter under environmentally relevant conditions. Our objectives were to (i) characterize spatial patterns of HgT accumulation; (ii) identify localized hotspots consistent with proximity to gold-trading infrastructure using probabilistic and geostatistical modeling; and (iii) evaluate the role of urban vegetation as a short- and long-term reservoir of vegetation-associated HgT in ASGM-impacted cities.

We show that HgT in urban vegetation exhibits spatial patterns consistent with proximity to gold trading infrastructure, and that the litter layer acts as a long-term terrestrial reservoir of vegetation-associated HgT. The localized spatial pattern observed suggests that tropical ASGM cities may function as plausible nodes of elevated HgT in vegetation within regional emission–transport pathways. These findings highlight the diagnostic potential of vegetation-based monitoring for screening ASGM-related Hg exposure and underscore the need for targeted environmental management strategies in Hg-impacted urban regions.

## 2 Materials and Methods

### 2.1 Study Area and Site Selection

The study was conducted in the Alta Floresta Mineral Province (AFMP), northern Mato Grosso State, Brazil, a region historically recognized as a global hotspot for Artisanal and Small-Scale Gold Mining (ASGM).^19,20^ The AFMP presents a tropical climate with distinct wet and dry seasons, typical of the Amazon biome transition zone.^21^ To evaluate vegetation-associated Hg loading under environmentally relevant conditions, three sites were selected to represent a controlled gradient of exposure (Figure 1):

1. Peixoto de Azevedo (PEI): an urban center with intense ASGM support activities and a high density of active gold-buying shops, representing the high-exposure scenario (Figure 1).
2. Nova Santa Helena (ASH): a nearby municipality with lower recorded ASGM activity, serving as a regional reference area (Figure 1E).
3. Native Forest Fragment (ANM): a preserved forest patch near PEI, used to establish baseline vegetation-associated HgT in minimally impacted vegetation (Figure 1D).

**Figure 1:**
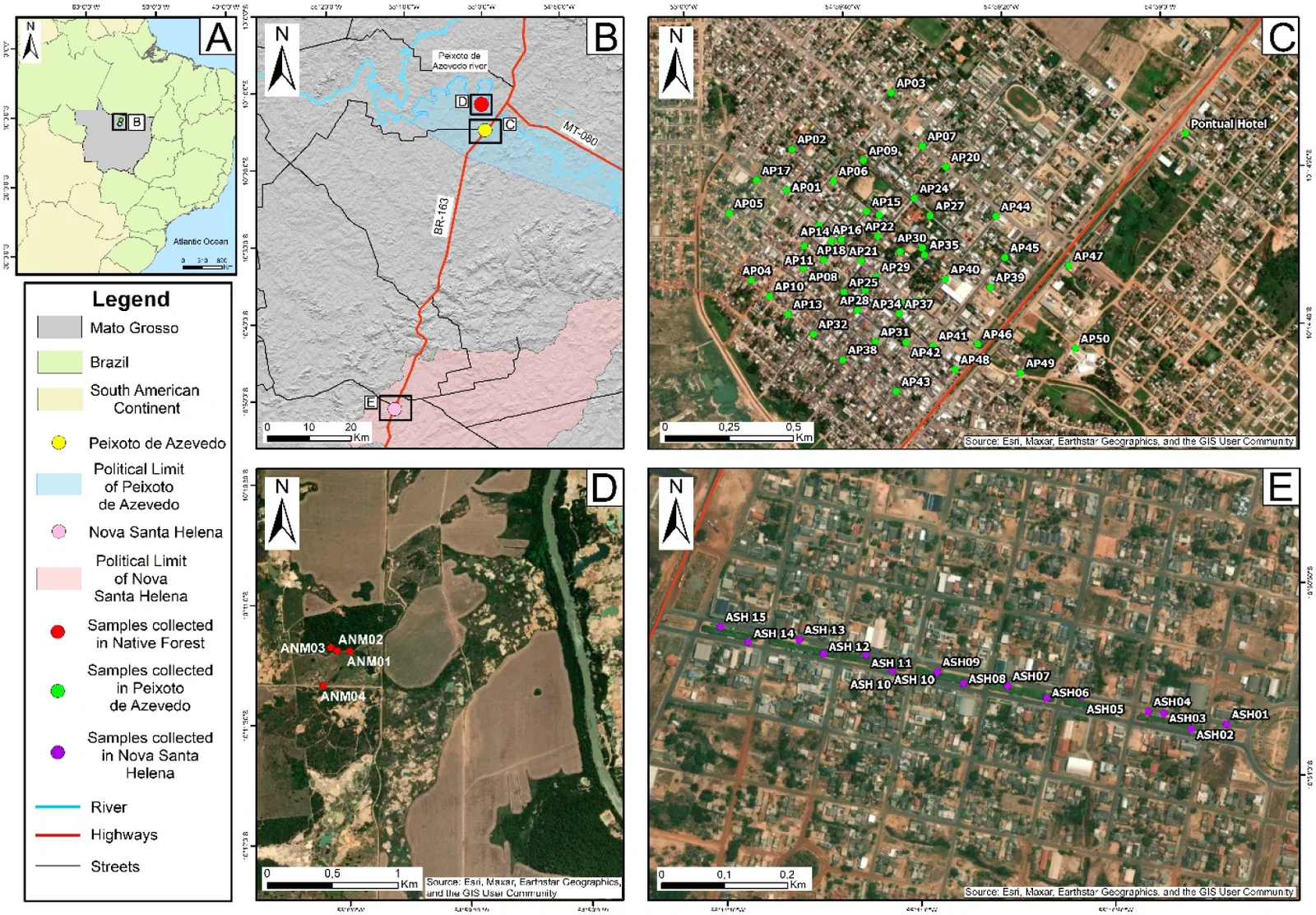
Study Area and Mercury Contamination Gradient in the Alta Floresta Mineral Province (AFMP), Brazil. (A) Location of the study area within Brazil and the South American Continent. (B) Regional map showing the municipalities of Peixoto de Azevedo (PEI) and Nova Santa Helena (ASH) relative to the main highways (BR-163 and MT-419). (C) Detailed view of the urban sampling grid in Peixoto de Azevedo, showing 50 sampling points (labeled AP) focused on the main commercial axis, where gold-trading activities are concentrated. (D) Location of the native forest control sites (ANM) near the active and inactive mining areas. (E) Detailed map of the urban sampling sites in Nova Santa Helena (ASH), the regional reference area. Base map and satellite imagery provided by Esri, Maxar, Earthstar Geographics, and the GIS User Community.

These sites collectively represent a gradient from lower-exposure forest vegetation (ANM) to low-exposure urban conditions (ASH) to high-exposure ASGM-impacted urban conditions (PEI). Sampling encompassed two vegetation compartments—canopy leaves and leaf litter—selected to capture both active atmospheric interception and postsenescence Hg accumulation.

### 2.2 Bioindicator Selection and Sampling Strategy

The common urban tree *Licania tomentosa* was selected as the biomonitor species due to its wide occurrence, uniform canopy structure, and documented capacity to accumulate HgT in tropical urban environments.^17^ A total of 79 samples were collected across three sites to investigate Hg partitioning and spatial loading: 50 leaf samples and 10 litter samples from PEI, 15 leaf samples from ASH, and leaf samples from four native tree species in the Native Forest Fragment (*Bauhinia angulata*, *Byttneria*, *Sloanea*, and *L. tomentosa*) (Figure 1).

Sampling was conducted during the dry season (August 2021) under stable meteorological conditions, with no rainfall in the preceding 72 h. Sampling points were defined on the basis of spatial analysis and proximity to potential emission sources. In PEI, a concentrated grid was established along Avenida Brasil, the main urban axis, targeting proximity to approximately 10 gold-buying shops, which were considered plausible localized Hg sources.

To standardize canopy sampling, four branches per tree were cut using a telescopic pruner at ∼3 m height, from which 10–15 mature leaves were composited. Leaves were not washed prior to analysis, following established biomonitoring protocols to preserve externally deposited material (including particulates and dust), while acknowledging that HgT also integrates internally assimilated Hg. Litter was collected manually directly beneath the crown projection, following criteria to ensure that only naturally senescent material was included and avoiding areas with recent pruning or displaced debris. Because paired canopy–litter sampling was possible only at 10 locations in PEI, the vertical comparison reflects conditions at these specific sites and cannot be generalized to ASH or ANM. In ASH, samples were collected along the main avenue with higher tree cover. All collected leaves were placed in plastic bags and immediately transported to the laboratory.

### 2.3 Total Mercury Determination

Samples were oven-dried at 60 °C to constant mass, manually cleaned of debris, and homogenized using stainless-steel scissors. Total mercury (HgT) concentrations were determined by the atomic absorption method using a Direct Mercury Analyzer (DMA-80 TRI-CELL; Milestone Inc., Italy), following validated protocols for plant material. The limit of detection (LOD) was 0.05 ng, and the limit of quantification (LOQ) was 0.16 ng of Hg.

### 2.4 Quality Assurance and Quality Control (QA/QC)

Analytical performance was verified daily by using certified reference materials (CRMs): apple leaves (NIST 1515), peach leaves (NIST 1547), and soil (NIST 2709). Recoveries ranged from 89.9% to 96.5% for the soil matrix. For low-concentration leaf matrices, daily recoveries ranged from 117% to 126%, representing a minor systematic instrument bias arising from cross-matrix quantification under the primary calibration file optimized for geological matrices. Although recoveries above 100% indicate an elevated positive bias in absolute HgT values, the uniformity of this bias across all samples ensures that relative spatial patterns, subpopulation segmentation, and ratio_C_ calculations remain valid for screening-level interpretation.

To preserve the operational integrity of the raw data set and avoid introducing secondary mathematical artifacts into the subsequent geostatistical interpolation and probabilistic subpopulation modeling, no empirical recovery correction factors were applied. Because this systematic bias was strictly uniform across all biological samples, it leaves the absolute spatial gradients, localized high-HgT zones, and vertical canopy-to-litter enrichment ratios (ratio_C_) unaffected, preserving their robustness for screening-level geochemical mapping. Precision was assessed through triplicate analyses of all samples, yielding a relative standard deviation (RSD) of <10%, well within the acceptance criteria for trace-level Hg determinations. Calibration curves (R^2^ > 0.999) were verified every 10 samples, and instrumental drift was monitored using midlevel calibration checks. Procedural blanks consistently showed a negligible amount of Hg, confirming the absence of laboratory contamination.

### 2.5 Spatial and Statistical Analysis

Data processing was conducted in Excel, calculating the average and standard deviation (SD) values for triplicate measurements. The normality of the data sets was assessed using the Shapiro-Wilk test (results provided in Table S1).

To assess the biogeochemical enrichment of mercury during foliar senescence and decomposition, we calculated the concentration ratio (Ratio_*C*_) between leaf litter and canopy leaves for each sampling point, defined as

$$RatioC=\frac{\left[HgT\right]Litter}{\left[HgT\right]CanopyLeaf}$$

Correlation between canopy and litter HgT was assessed using Pearson and Spearman coefficients (Table S3; Figure S2).

Differences between PEI and ASH were evaluated using the Mann–Whitney U test due to non-normality and unequal variances, and confirmed using Welch’s *t*-test on log-transformed data (Table S3). 95% confidence intervals for mean HgT values were obtained using one-sample *t*-tests (Table S2).

To identify statistically distinct subpopulations within the Hg data set, we applied probabilistic analysis based on the Sinclair method, using PPlot software.^22^ This approach enabled the segmentation of the log-transformed data into two linear trends, corresponding to high-concentration and low-concentration subpopulations, as confirmed by probability plots. Spatial interpolation of HgT concentrations was performed by using ordinary kriging in ArcGIS 10.8. Interpolated surfaces were generated for canopy leaves and leaf litter to visualize spatial gradients and identify localized HgT hotspots that are spatially consistent with ASGM-related point sources. Comparative spatial analysis between PEI and ASH was used to establish a regional baseline, with ASH serving as a low-exposure reference due to the absence of gold-trading infrastructure.

Variogram modeling and cross-validation statistics are provided in Table S4. In Peixoto de Azevedo (PEI), a Gaussian variogram model was fitted, characterized by a high nugget (23 422.5) and strong small-scale variability, reflecting the presence of localized hotspots. Due to this pronounced microscale heterogeneity, the model was retained for exploratory visualization only, and kriging predictions should be interpreted with caution. In Nova Santa Helena (ASH), an exponential variogram model provided a stable spatial structure, with a low cross-validation error (RMSE = 35.28 μg kg^–1^) and minimal bias, supporting its suitability for exploratory interpolation. Variogram modeling was not performed for the native forest fragment (ANM) because the sample size (n = 4) was insufficient to characterize spatial dependence.

Alternative variogram models (spherical, exponential) were tested but did not improve cross-validation performance, reinforcing that the Gaussian model is suitable only for exploratory visualization. Interpolation uncertainty is elevated due to the high nugget effect, and the ∼300 m spatial range should therefore be interpreted as an approximate pattern rather than a precise plume boundary.

## 3 Results

### 3.1 Regional Baseline and the Hg Exposure Gradient

HgT concentrations in the regional reference sites define a low-exposure baseline for the northern Mato Grosso region. In the native forest fragment (ANM), concentrations showed species-specific variation, ranging from 16.84 ± 0.3 μg kg^–1^ in *Licania tomentosa* to 89.75 ± 0.9 μg kg^–1^ in *Bauhinia angulata* (Table 1). These values define the range of HgT concentrations observed in native vegetation at the forest fragment.

**Table 1:** Mean Total Mercury (HgT) Concentrations in Leaves of Native Tree Species from the Forest Control Site Near PEI

**Species**	Sampling Point	Mean HgT (μg kg^–1^) ± SD
*Bauhinia angulata*	ANM 01	89.75 (± 0.9)
*Byttneria*	ANM 02	41.4 (± 3.2)
*Sloanea*	ANM 03	39.58 (± 0.7)
*Licania tomentosa*	ANM 04	16.84 (± 0.3)

Table showing the Total Mercury (HgT) concentrations (μg kg^–1^) measured in the leaves of four native tree species collected in the Native Forest Fragment near PEI. Values indicate species-specific differences in measured HgT concentrations, with *Bauhinia angulata* showing the highest mean value (89.75 μg kg^–1^). Concentrations are compared against the HgT value for *Licania tomentosa* in the same fragment. All mean concentrations are reported with their respective standard deviations (SD).

Similarly, in the reference municipality of Nova Santa Helena (ASH), concentrations were consistently low across sampling sites (Figure 2). The mean HgT concentration in ASH was 20.58 μg kg^–1^ (SD = 32.11 μg kg^–1^; median = 10.81 μg kg^–1^), as detailed in the Supporting Information (Table S1). The majority of the sampling sites fell within the baseline interval of 6.82–35.31 μg kg^–1^, with the exception of a localized higher concentration at site ASH-11, which reached 135.24 μg kg^–1^ (Figure 2). Despite this local variation, a predominantly unimodal distribution pattern in ASH is supported by the normal probability plot (Figure 3A) and the frequency histogram (Figure S1A), consistent with distribution characteristics typical of low-exposure conditions.

**Figure 2:**
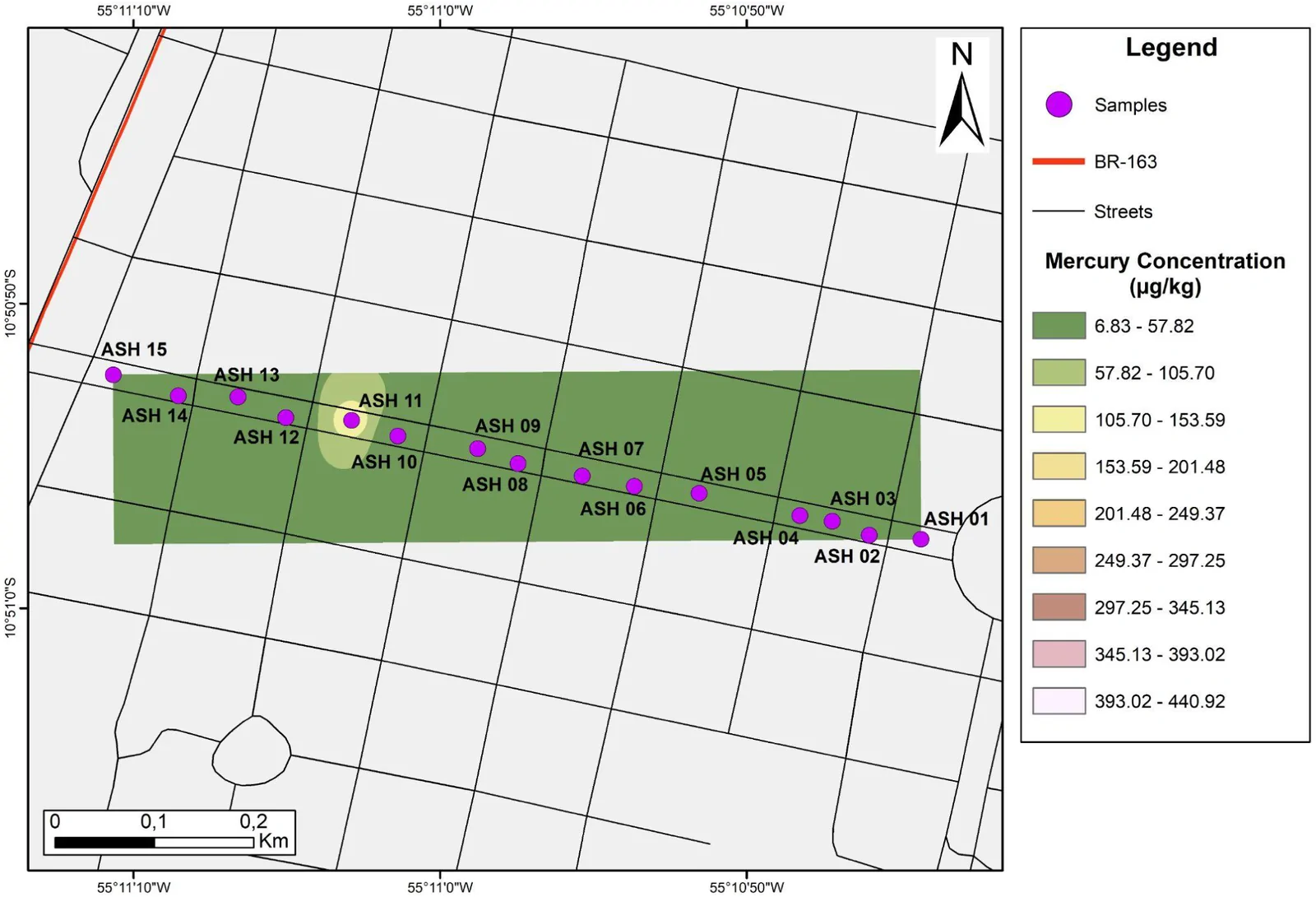
Regional Hg Baseline and Exposure Gradient in Nova Santa Helena (ASH). Map of total mercury (HgT) concentrations in *Licania tomentosa* canopy leaves across the reference municipality. The majority of sites define a consistent regional baseline (6.82–35.31 μg kg^–1^), with a single localized higher concentration at ASH-11 (135.24 μg kg^–1^). To facilitate direct visual comparison of mercury loading between study areas, the color scale is standardized to the urban impact range observed in Peixoto de Azevedo. Color classes represent HgT concentration intervals (μg kg^–1^).

**Figure 3:**
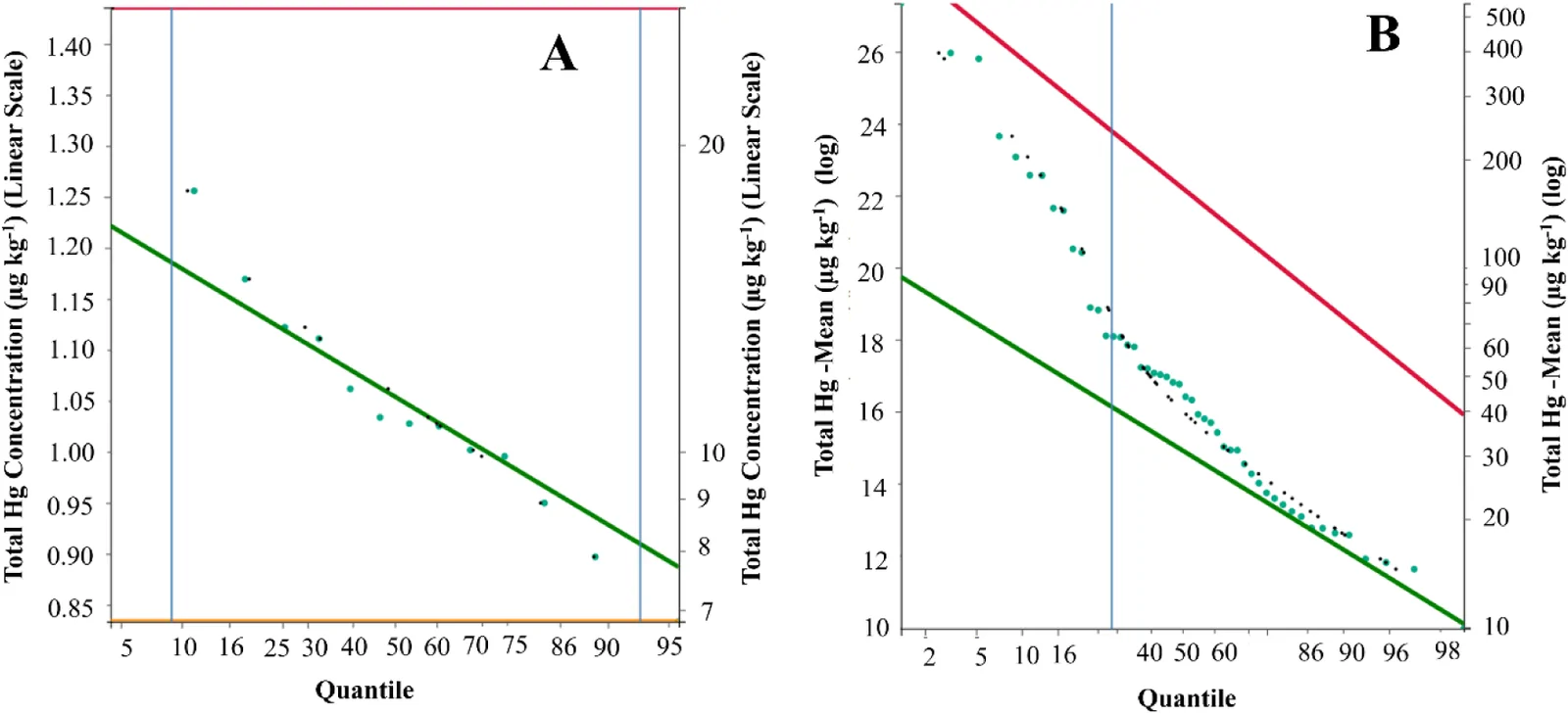
Comparative probabilistic analysis of total mercury (HgT) concentrations in *Licania tomentosa* leaves. (A) Probability plot for the regional reference site, Nova Santa Helena (ASH), displaying a single linear trend consistent with a unimodal, normally distributed data set. (B) Probability plot for Peixoto de Azevedo (PEI), with axes in log scale, showing two distinct linear segments: the steeper upper segment (red) corresponds to Subpopulation 1; the shallower lower segment (green) represents Subpopulation 2. The vertical blue line marks the statistical cutoff separating the two groups.

### 3.2 High-Resolution Spatial Hg Loading in the Urban Center (Peixoto de Azevedo)

In contrast to the regional baseline, high-resolution sampling in the urban center of Peixoto de Azevedo (PEI) revealed a broad log-normal distribution of HgT concentrations in *Licania tomentosa* canopy leaves, ranging from 9.92 μg kg^–1^ to 543.10 μg kg^–1^ (Table S1). Probabilistic analysis using the Sinclair method (Figure 3B) successfully segregated the data set into two statistically distinct subpopulations (Table 2): a high-concentration group (Subpopulation 1), with a modeled range reaching 607 μg kg^–1^, comprising 28.67% of the samples, and a low-concentration group (Subpopulation 2), representing 71.33% of the data set, with a modeled range between 11.53 and 45.62 μg kg^–1^ (mean = 31.04 μg kg^–1^) (Table 2).

**Table 2:** Statistical Characteristics of Hg Sub-Populations in *Licania tomentosa* Leaves (Peixoto de Azevedo) Identified by PPlot Modeling^a^

Subpopulation	Interval (1σ) (μg kg^–1^)	Quantity (n%)	Mean (μg kg^–1^)	Standard Deviation (1σ) (μg kg^–1^)
High Hg (1)	83.55–607	28.67	166	151
Intermediate (1 + 2)	45.62–83.55	-	-	-
Low Hg (2)	11.53–45.62	71.33	31.04	19.88

a Intervals and statistical parameters represent modeled subpopulations; for raw minimum and maximum values, see Table S1 in Supporting Information.

The linear segmentation observed in the log-probability plot (Figure 3B) confirms the sharp statistical separation between these two groups. While Subpopulation 1 corresponds to the upper tail of the distribution, Subpopulation 2 reflects the lower-concentration range within the urban perimeter, which is higher than the median values observed at the ASH reference site (Table S2). Importantly, the interval 11.53–45.62 μg kg^–1^ corresponds to the modeled low-Hg subpopulation within PEI and does not represent regional background conditions. The overall deviation from normality in PEI canopy leaves was confirmed by the Shapiro-Wilk test (p = 0.004; Table S1) and is visualized in the skewed frequency histogram (Figure S1B).

### 3.3 Spatial Distribution of Urban Hg Loading

Exploratory spatial interpolation of total mercury (HgT) concentrations in canopy leaves (Figure 4) highlights prominent local HgT hotspots concentrated along the main commercial axis—Avenida Brasil. Although geostatistical modeling captured a broad regional range (α = 5,275.4 m; Table S4), reflecting widespread urban baseline elevation, the visual surface is strongly influenced by intense microscale heterogeneity (Nugget = 23,422.5). This results in localized peaks where the highest HgT values (>393.02 μg kg^–1^) occur within a radius of approximately 300 m around the active gold-buying shops identified in the urban grid (Figure 4).

**Figure 4:**
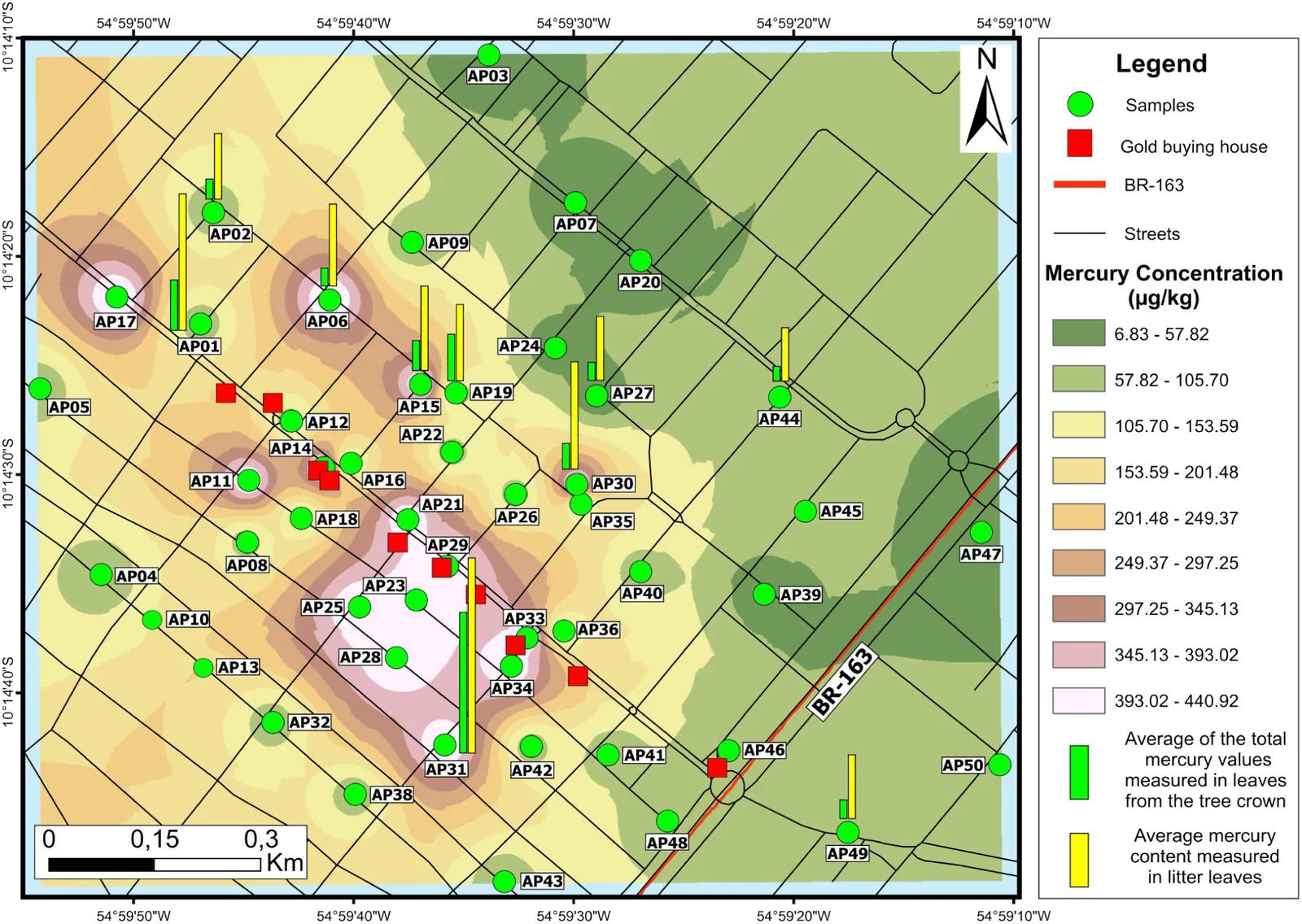
Spatial Distribution of HgT and Biogeochemical Partitioning in Urban Peixoto de Azevedo (PEI). The exploratory interpolated surface based on canopy leaf measurements highlights prominent local HgT zones concentrated near gold-buying shops (red squares), superimposed on a high-heterogeneity baseline (Gaussian model, Nugget = 23,422.5). Bar graphs compare HgT concentrations in canopy leaves (green) and leaf litter (yellow), showing higher measured values in the litter layer (Ratio_C_ ≈ 3.15).

Both vegetation compartments exhibited coupled distribution patterns, anchoring the core areas of high accumulation. Canopy leaves (green bars) define the core of the interpolated high-HgT zone. Leaf litter samples (yellow bars), although excluded from the interpolation modeling due to sample size constraints (n = 10), consistently displayed higher absolute HgT values than their paired canopy counterparts at the most-impacted sites, such as AP31 (203.11 ± 14.1 μg kg^–1^ in leaf litter vs 146.32 ± 7.1 μg kg^–1^ in canopy; Table 3). Across the municipal grid, HgT concentrations in the leaf litter followed the exact same spatial gradient observed in the canopy, with absolute values decreasing substantially at distal sampling points farther away from the commercial corridor (Figure 4).

**Table 3:** Partitioning of Total Mercury (HgT) in Canopy Leaves and Leaf Litter of *Licania tomentosa* in Peixoto de Azevedo (PEI)^a^

	Mean HgT (μg kg^–1^) ± SD		
Sampling Point	Canopy Leaves	Leaf Litter	Ratio_C_
AP 01	52.39 (± 2.2)	142.29 (± 14.4)	2.72
AP 02	20.32 (± 2.7)	68.02 (± 9.2)	3.35
AP 06	18.06 (± 0.7)	84.82 (± 4.7)	4.70
AP 15	31.09 (± 1.2)	87.26 (± 2.7)	2.81
AP 19	47.97 (± 1.1)	79.22 (± 4.4)	1.65
AP 27	18.29 (± 1.4)	66.08 (± 7.3)	3.61
AP 30	26.71 (± 1.1)	111.87 (± 4.5)	4.19
AP 31	146.32 (± 7.1)	203.11 (± 14.1)	1.39
AP 44	15.14 (± 0.9)	55.31 (± 4.9)	3.65
AP 49	18.89 (± 2.2)	66.40 (± 10.1)	3.51

a Table detailing the average Total Mercury (HgT) concentrations (μg kg^–1^) for paired samples of canopy leaves and leaf litter collected at the same sites in PEI. The table also reports the Concentration Ratio (Ratio_C_), calculated as [HgT]­Litter/[HgT]­Canopy Leaf. Standard deviation (SD) values are provided for all mean concentrations.

Comparative analysis showed consistently higher HgT concentrations in leaf litter than in canopy leaves across all paired points. HgT concentrations in the leaf litter were higher than those in the actively intercepting canopy leaves across all paired points (Table 3). Quantification of this biogeochemical enrichment via the Concentration Ratio (Ratio_C_ = [HgT]_Litter_/[HgT]_Canopy Leaf_) showed a vertical gradient ranging from 1.39 (at the highly impacted site AP31) to 4.70 (at site AP06), yielding a grid-wide average of 3.15 ± 0.9 (Table 3; Table S3). This robust positive relationship between the two urban vegetation compartments was mathematically validated by coregistered parametric and nonparametric correlation metrics, showing a high level of significance (Figure S2; Table S3: Pearson r = 0.91, p = 0.00025; Spearman p = 0.81, p = 0.008).

## 4 Discussion

### 4.1 Regional Baseline, Species Traits, and Natural Drivers of Hg Retention

The markedly lower HgT concentrations observed in ASH and in the ANM establish a robust regional baseline. In ASH, although the mean concentration is 20.57 μg kg^–1^, the median value of 10.81 μg kg^–1^ more accurately represents the local Hg levels due to the influence of a localized outlier (Table S1). This median is substantially lower than typical foliar Hg levels reported for Amazonian rainforest vegetation, which generally range from 20 to 50 μg kg^–1^,^11,23^ reinforcing the suitability of ASH as a high-quality regional reference site. The ASH data set conforms to a single, normally distributed population (Figure S1A; Table S1), indicating statistical homogeneity across the area. In contrast, the elevated and heterogeneous concentrations observed in Peixoto de Azevedo (PEI) reveal a pronounced regional Hg exposure gradient (Figure S1B), consistent with proximity to gold-buying shops and ASGM-related activities —activities entirely absent in ASH.^6,7,13^

The isolated high-concentration point at ASH 11 (135.24 ± 19.8 μg kg^–1^) warrants attention as a localized outlier. Its proximity to the BR-163 corridor (Figure 1E) suggests that regional transport may facilitate Hg vapor deposition from larger ASGM centers. Despite this outlier, the median of 10.81 μg kg^–1^ reinforces that, in the absence of direct gold-trading sources, regional Hg loading remains at baseline levels.^17^ *L. tomentosa* remains the most suitable species for standardized monitoring due to its widespread urban distribution and consistent canopy structure, supporting broader applications of vegetation-based monitoring in ASGM-impacted regions.^5,9,24,25^ This reliability of urban broadleaved species for tracking vegetation-associated HgT patterns, despite variations in physiological traits, aligns with recent urban dendrochemistry findings in Europe,^26^ confirming that foliar compartments are highly sensitive diagnostic tools for screening spatial HgT patterns in urban environments.

Within ANM, the differences in Hg retention among species (Table 1) highlight the role of foliar traits in vegetation-associated HgT accumulation. *Bauhinia angulata* exhibited the highest measured concentration (∼89.75 μg kg^–1^), but because each species is represented by a single sample, this observation cannot be interpreted as superior Hg capture capacity. Its foliar traits—surface roughness and simple tector trichomes—likely enhance Hg deposition. Although foliar interception is a transient sink, the elevated levels in *B. angulata* relative to *L. tomentosa* highlight how specific leaf area and surface roughness may influence vegetation-associated HgT in tropical forest fragments. These species-specific differences also help interpret the variability observed in PEI, where functional traits may modulate Hg interception across the urban grid.

### 4.2 Mechanisms of Urban Hg Contamination and the Influence of Point Sources

Having established the regional baseline and the natural drivers of foliar Hg retention, we now examine the mechanisms responsible for urban Hg contamination in PEI. High HgT levels in PEI correlate directly with specific gold-buying houses, suggesting a plausible influence of these stationary point sources. The wide, log-normal distribution of Hg in *L. tomentosa* leaves supports the use of a probabilistic model (Figure 3B) to segregate the data into two distinct populations. Sub population 1 (Impacted Sub population; mean = 166 μg kg^–1^) represents a vegetation-associated HgT pattern consistent with localized atmospheric influence, with its spatial extent coinciding with the commercial axis where gold-buying shops are concentrated. The probabilistic approach minimizes subjective thresholding and provides a statistically grounded separation of impacted and non-impacted samples.

This pattern provides indirect evidence consistent with elevated HgT near gold-trading areas, although direct atmospheric measurements would be required to confirm emission fluxes, specifically through the release of gaseous elemental mercury (GEM) during secondary smelting of Au–Hg amalgams. It is important to note that our data set does not resolve Hg speciation and therefore cannot confirm whether GEM is the dominant atmospheric Hg form contributing to the observed vegetation-associated HgT patterns. Future natural-abundance Hg stable isotope analysis would provide a critical next step to resolve source contributions and validate the spatial interpretation presented here.

Our results corroborate elevated atmospheric Hg loads associated with ASGM activities in the Amazon^5,10^ and align with passive sampling and biomonitoring studies that identified GEM hotspots near gold-related infrastructures.^9,24^ Furthermore, this explicit trend of Hg concentration gradients declining progressively from the inner urban center toward peripheral reference areas has been recently validated by high-resolution grid-based models in other major global cities.^27^ High-resolution mapping shows that although the urban grid is heterogeneously impacted, the most severe contamination is restricted to the immediate vicinity of gold trading facilities, consistent with findings from other ASGM-impacted cities.^8,28,29^

Geostatistical modeling revealed an elevated nugget effect (C_0_ = 23,422.5 in PEI; Table S4), representing intense microscale spatial heterogeneity superimposed on a broad regional range (α = 5,275.4 m). This indicates that while diffuse atmospheric transport elevates background levels across the wider urban grid, the dense canopy near emission sources drives rapid, localized interception. This dual spatial behavior explains why the highest Hg concentrations form sharp, intense hotspots in the immediate vicinity of gold-trading units, despite the extensive mathematically modeled range. Identifying that 28.67% of urban samples belong to the impacted subpopulation (Table 2) allows precise quantification of this localized spatial pattern within the broader urban baseline.

### 4.3 Biogeochemical Partitioning, Monitoring Implications, and Global Relevance

The consistently higher HgT concentrations in leaf litter compared to canopy leaves (Figure 4; Table S1) reveal that the litter layer shows evidence of acting as a persistent terrestrial reservoir for vegetation-associated HgT within the sampled PEI sites. This enrichment, quantified in Table 3, is consistent with prolonged exposure and preferential Hg retention relative to carbon loss during senescence, as suggested in previous studies.^10,30,31^ According to Zhou et al.,^23^ over 90% of Hg in foliage is integrated into internal tissues, where it is oxidized and bound to sulfur-containing ligands, such as thiols (e.g., cysteine residues). Such internal sequestration has been proposed as a mechanism contributing to Hg stability in senescent tissues, although our data set cannot directly confirm these processes.^23^

This enrichment process may be influenced under the unique biogeochemical conditions of the Southern Hemisphere tropics—characterized by high turnover rates and specific vegetation types.^12^ The leaf litter can accumulate HgT, where the degradation of structural polymers exposes new binding sites that sequester both dry-deposited Hg and Hg washed from the canopy. The Concentration Ratio (Ratio_C_) averaged ∼3.15 across sites, reaching 4.70 at AP 06.

In PEI, the fate of Hg-enriched litter is shaped by both natural and anthropogenic pathways. While part of the litter decomposes in situ—transferring Hg to the soil-surface interface and associated food webs—another portion is removed through municipal sweeping, creating a secondary transport pathway in which sequestered Hg may be redistributed within the city’s waste system. In ASGM hubs, where litter concentrations reached a measured maximum of 203.11 μg kg^–1^ (corresponding to areas where modeled canopy concentrations reached 607 μg kg^–1^), urban leaf litter effectively transitions from a natural byproduct to a concentrated sink of vegetation-associated HgT.

These findings reinforce the relevance of the regional baseline observed in ASH (6.82–35.31 μg kg^–1^), which represents vegetation-associated HgT levels in the absence of gold-trading infrastructure.^33^ Within this context, the differences in Hg retention observed in the ANM fragment suggest that functional traits—such as surface roughness and simple tector trichomes—may influence vegetation-associated HgT, although this observation is preliminary given the limited sample size. *Bauhinia angulata* showed a higher measured HgT value in this single sample, but this cannot be interpreted as superior Hg-capture capacity without replicated species-level data. Evidence from the broader genus suggests that *Bauhinia* species may capture airborne contaminants, but this remains a hypothesis requiring replication in the context of HgT.^34^

Urban tree composition further dictates the potential for contaminant filtering. In many municipalities of Mato Grosso, urban forestry is dominated by *Licania tomentosa*, resulting in low functional diversity.^18,35^ These observations raise the hypothesis that functional diversity may influence vegetation-associated HgT patterns, although further replicated studies are required. Increasing species diversity has been proposed as a strategy to enhance pollutant interception, a priority in South America, where foliage Hg concentrations are among the highest in the world.^23^ Incorporating native taxa with diverse functional traits may enhance the robustness of vegetation-based monitoring frameworks. Integrating these with other groups, such as epiphytic bromeliads (*Tillandsia* spp.), offers a resilient framework for vegetation-based monitoring in Hg-impacted urban landscapes.^3,5,24^

Although our study did not quantify atmospheric Hg fluxes or litterfall mass, the observed HgT concentrations allow a qualitative assessment of ecological significance. Assuming typical tropical litterfall rates of 5–10 Mg ha^–1^ yr^–1^ reported for Amazonian forests,^1,11^ even moderate HgT values (50–150 μg kg^–1^) correspond to potential annual Hg inputs on the order of 0.25–1.5 mg Hg m^–2^ yr^–1^, which align with ranges reported for tropical forest ecosystems.^11,23^ The elevated litter concentrations observed at PEI (up to 203.11 μg kg^–1^ at site AP 31; Table 3) suggest that urban vegetation may intercept and transfer non-negligible Hg masses to surface organic layers in ASGM-impacted cities. These estimates are approximate and intended only to contextualize the environmental relevance of the measured concentrations; direct flux measurements remain necessary for quantitative confirmation.

### 4.4 Environmental Justice, Regulatory Gaps, and Implications for ASGM-Impacted Cities

High-resolution mapping of Hg in PEI highlights not only the spatial dynamics of contamination but also the spatial proximity of elevated HgT to areas frequented by nonmining urban populations. This may have implications for exposure among residents who are not directly involved in mining activities. Similar patterns have been documented in other ASGM regions, such as Ghana, where nonmining households near ASGM corridors exhibit proximity-related exposure concerns.^36^ In low- and middle-income countries (LMICs), where regulatory gaps and informal economies may influence environmental oversight, mercury remains a priority pollutant identified as a pollutant of concern.^15,37–40^

In Brazil, the regulatory landscape reflects these global gaps. While CONAMA Resolution No. 396/2008 ^41^ and ANVISA Normative Instruction No. 160/2022 ^42^ establish limits for Hg in water and food, no standards exist for Hg in nonedible plant tissues, despite their widespread use in environmental monitoring. Technical reports, such as the Regime Jurídico do Mercúrio no Brasil,^43^ confirm that national legislation remains centered on industrial effluents, with limited coverage of vegetation-based indicators in urban ASGM hubs.

The regional baseline observed in ASH (6.82–35.31 μg kg^–1^) provides a reference for interpreting vegetation-associated HgT patterns in ASGM-impacted cities. The use of native tree species as passive samplers is particularly relevant in ASGM regions of West Africa, Southeast Asia, and Latin America, where monitoring infrastructure is limited.^14^ The relevance of such low-cost monitoring is underscored by the shorter atmospheric lifetime of Hg (3–6 months) recently reported, which implies that Hg is largely a hemispheric pollutant. As highlighted by Schneider et al.,^12^ the Southern Hemisphere faces persistent data gaps in long-term Hg monitoring despite its unique biome heterogeneity and increasing ASGM emissions. Establishing this baseline in the Amazon contributes to improving geographic coverage in regional Hg assessments. Vegetation-based biomonitoring may complement existing environmental assessment tools in ASGM-impacted regions.^44^ Any suggestion regarding the potential use of *Bauhinia angulata* in urban buffer zones should be considered preliminary, as our data set contains only one sample per species and cannot establish trait-based differences in Hg interception.

This context is reinforced by recent global assessments. As highlighted by Ahmad and Ali,^45^ discussions at COP6 of the Minamata Convention emphasized that prohibiting mercury use alone has not curbed emissions in many ASGM regions; instead, restrictions without viable economic alternatives have pushed amalgam burning into informal and poorly regulated urban spaces. The localized HgT patterns observed in PEI are consistent with reports of informal amalgam processing occurring in urban settings, underscoring the potential value for vegetation-based surveillance and community-level monitoring where formal regulatory controls are limited.

### 4.5 Environmental Implications and Limitations

#### 4.5.1 Methodological and Environmental Limitations

While this study provides a high-resolution, robust framework for tropical urban biomonitoring, specific limitations linked to complex anthropized and deforested landscapes must be noted:

**Urban Leaf Litter Availability (N = 10):** Paired canopy-to-litter sampling was restricted to 10 coregistered locations across the PEI grid. In active urban centers, continuous municipal sweeping and public street cleaning services systematically remove fallen biomass to maintain city hygiene. This intensive human management limits the accumulation of stable litter layers under urban trees. Nevertheless, the strong statistical consistency of the coregistered parametric and nonparametric correlations (Pearson *r* = 0.91, Spearman *p* = 0.81; Table S3) demonstrates that this sample size was adequate to characterize the canopy-to-litter vertical partitioning gradient, successfully detecting Hg enrichment trends even where biomass accumulation is heavily disrupted by municipal services.

**Forest Fragment Sample Size (N = 4):** Sampling within the regional background native forest fragment (ANM) was limited to four dominant target species. This constraint reflects the severe environmental reality of the southern Amazonian “Arc of Deforestation”, where historic and ongoing gold-mining expansions have left native forest ecosystems highly fragmented, diminished, and isolated. Despite the restricted number of native taxa evaluated, these samples provided a useful ecological baseline, confirming that interspecific functional traits (such as the trichomes of *Bauhinia angulata*) may influence vegetation-associated HgT, although this observation is preliminary.

**Analytical Cross-Matrix Calibration Bias:** Low-concentration plant tissues (canopy leaves and leaf litter) were quantified under an operational primary calibration curve optimized for geological matrices. While this cross-matrix approach introduced a systematic instrument bias—evidenced by elevated recoveries (117–126%) for the NIST 1515 leaf standard—the bias was strictly uniform across all biological samples. Consequently, while the absolute values may represent a conservative upper bound estimate, the relative spatial gradients, the identification of localized hotspots, and the vertical canopy-to-litter enrichment ratios (Ratio_C_) remain entirely robust and unaffected. This operational choice ensures data set integrity by avoiding secondary mathematical artifacts that could arise from empirical normalization factors in complex geostatistical and probabilistic subpopulation models.

#### 4.5.2 Environmental Implications and Global Governance

The vegetation-associated HgT patterns observed in PEI may have implications for biomass handling and urban waste pathways. Under severe exposure near gold-buying shops, where litter concentrations reached a maximum of 203.11 μg kg^–1^, urban leaf litter effectively transitions from a benign organic byproduct into a concentrated sink of vegetation-associated HgT. If municipal waste management routes this biomass into conventional open landfills or municipal composting systems, it may redistribute the sequestered Hg into soils and groundwater, or facilitate re-emission through surface legacy degassing.^23^ This emphasizes that urban trees can accumulate HgT^32^ but they highlight the need to consider biomass management in Hg-impacted urban settings.

Furthermore, our results suggest that the functional traits of urban vegetation may influence HgT patterns. Functional traits observed in *Bauhinia angulata* raise the hypothesis that certain native taxa may offer complementary monitoring potential, although species-level replication is needed before recommending their use in targeted buffer zones.^27,32^ This strategy is potentially relevant for ASGM-affected towns across Latin America, Africa, and Southeast Asia, where formal regulatory oversight of informal gold trading is economically limited.^45^ Utilizing established urban trees as low-cost passive bioindicators provides a practical approach to complementing existing environmental assessment tools in ASGM-impacted regions.

## 5 Conclusions

This study provides a high-resolution assessment of vegetation-associated total mercury (HgT) across an ASGM-impacted tropical urban center and two reference sites. We identified a statistically distinct high-HgT subpopulation in Peixoto de Azevedo (PEI) and observed consistent HgT enrichment in leaf litter relative to canopy leaves at sampled PEI locations. Spatial patterns of HgT were consistent with proximity to gold trading infrastructure, although direct atmospheric measurements would be required to confirm emission sources or fluxes. These findings support the use of *Licania tomentosa* as a practical biomonitor for screening Hg exposure in ASGM-impacted cities, and highlight the need for future work, including atmospheric Hg measurements, Hg speciation and isotopes, species-level replication to test functional trait effects, replicated litter sampling in reference areas, and quantitative flux estimates to fully resolve sources and mechanisms.

## Supplementary Material



## Data Availability

Data will be made available upon request.

## Notes

Frequency histograms of HgT concentrations (Figure S1); canopy–litter correlation analysis (Figure S2); descriptive statistics for all sampling compartments (Table S1); confidence intervals for HgT means (Table S2); comparative statistical tests and enrichment ratios (Table S3); and variogram models and cross-validation metrics for spatial interpolation (Table S4) (PDF)

## ⊥

Department of Geology, Federal University of Ceará (UFC), Campus do Pici, Bloco 912, CEP 60440-554, Fortaleza, Ceará, Brazil
