# Supplementary material for: Vegetation-Associated Mercury Patterns and Biogeochemical Signals in a Tropical Urban Mining Region
Source: ACS Omega. 2026 Oct 17;11(43):67185–95. doi: 10.1021/acsomega.6c07837 (PMC13737974; doi:10.1021/acsomega.6c07837)
Supplement: Supplementary file 1 [file ao6c07837_si_001.pdf]

SUPPORTING INFORMATION

Vegetation-Associated Mercury Patterns and Biogeochemical Signals in a Tropical Urban Mining Region

Jerusa Schneider[1*], Sheyla dos Santos Brito[1], André Massanobu Ueno Kunifoshita[1 †], Ricardo Lopes Tortorela de Andrade[2], Anne-Hélène Fostier[3], James Lambert-Smith[4], Maria José Maluf de Mesquita[1]

[1] Department of Geology and Natural Resources (DGRN), Institute of Geosciences, University of Campinas (UNICAMP), Rua Carlos Gomes, 250, CEP 13083-855, Campinas, SP, Brazil

[2] Graduate Program in Environmental Sciences, Institute of Natural, Human and Social Sciences, Federal University of Mato Grosso (UFMT), Av. Alexandre Ferronato, 1200, CEP 78557-267, Sinop, MT, Brazil

[3] Department of Analytical Chemistry, Institute of Chemistry, University of Campinas (UNICAMP), P.O. Box 6154, CEP 13083-970, Campinas, SP, Brazil

[4] School of Earth and Environmental Sciences, Cardiff University, Main Building, Park Place, CF10 3AT, Cardiff, Wales, UK

[†]**Present Address:** Department of Geology, Federal University of Ceará (UFC), Campus do Pici, Bloco 912, CEP 60440-554, Fortaleza, Ceará, Brazil

***Corresponding author:** Jerusa Schneider - Department of Geology and Natural Resources (DGRN), Institute of Geosciences, University of Campinas (UNICAMP), Rua Carlos Gomes, 250, CEP 13083-855, Campinas, SP, Brazil; Email: (jerusasc@unicamp.br)

Figure S1. Frequency distribution of HgT concentrations in *Licania tomentosa* leaves.

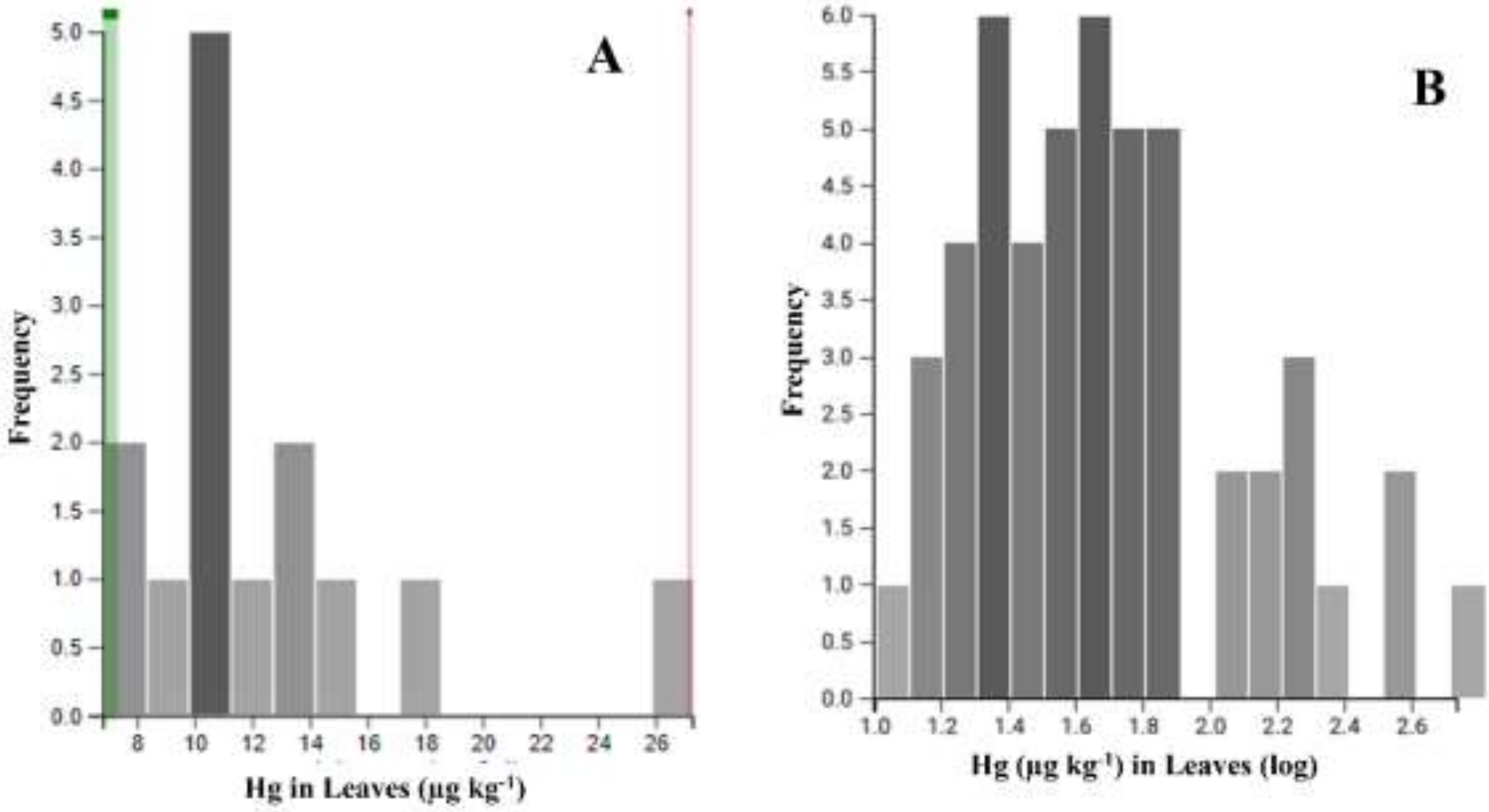

(A) Histogram of total mercury (HgT) concentrations in *Licania tomentosa* leaves from Nova Santa Helena, showing a unimodal and nearly symmetric distribution consistent with low-exposure conditions.
(B) Histogram of log-transformed HgT concentrations in *Licania tomentosa* leaves from Peixoto de Azevedo, showing a skewed distribution with deviation from unimodality, indicative of heterogeneous exposure patterns.

Figure S2. Linear regression and correlation analysis between total mercury (HgT) concentrations (µg kg^{-1}) in canopy leaves and paired leaf litter samples from Peixoto de Azevedo (n = 10)

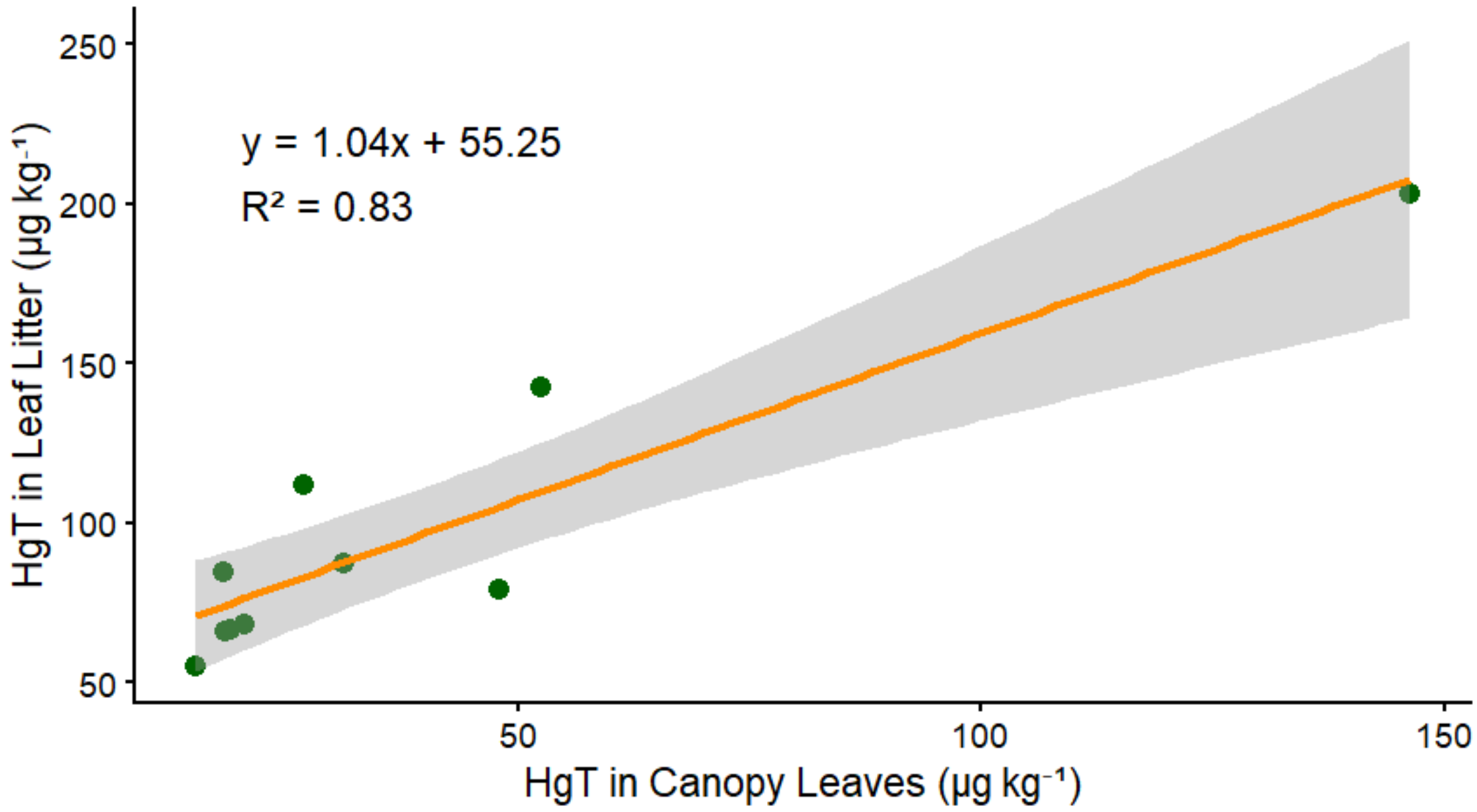

The solid orange line represents the linear regression fit (y = 1.04x + 55.25; R^2 = 0.83). The shaded gray area indicates the 95% confidence interval for the regression line. Green dots represent individual coupled sampling points, highlighting the strong positive relationship between canopy interception and HgT accumulation in litter.

Table S1. Descriptive statistics of total mercury (HgT) concentrations (μg kg^{-1}) in canopy leaves and leaf litter across the different study sites.

Statistical Data	Peixoto de Azevedo (Canopy)	Leaf Litter (Peixoto)	Nova Santa Helena (Reference)	Native Forest (Control)
**Number of samples (n)**	50	10	15	4
**Mean**	81.93	96.44	20.58	46.89
**Median**	45.59	82.02	10.81	40.49
**Maximum**	543.10*	203.11	135.24	89.75
**Minimum**	9.92	55.31	6.82	16.84
**Standard Deviation**	106.95	45.40	32.11	30.68
**Shapiro-Wilk (p-value)**	0.004	0.373	0.167	0.9586

*For Peixoto de Azevedo (Canopy), the raw measured range (9.92 – 543.10 μg kg^{-1}) encompasses the overall modeled sub-population interval (11.53 – 607 μg kg^{-1}, Table 2) identified via PPlot software, spanning from the low-exposure (11.53 – 45.62 μg kg^{-1}) to the high-exposure sub-population (83.55 – 607 μg kg^{-1}).

Table S2. Statistical summary and 95% confidence intervals (CI) for mean total mercury (HgT) concentrations across different sampling compartments and study sites.

Study Site	Mean (μg kg^{-1})	95 % CI	Median	Min–Max
**PEI (canopy)**	81.93	51.66–112.20	45.59	9.92–543.10
**ASH (canopy)**	20.58	2.80–38.36	10.81	6.82–135.24
**Leaf Litter**	96.44	63.95–128.92	82.02	55.31–203.11
**Native forest (ANM)**	46.89	—	40.49	16.84–89.75

95% confidence intervals were calculated using one-sample t-tests to evaluate the statistical boundaries of Hg loading. PEI = Peixoto de Azevedo (high exposure); ASH = Nova Santa Helena (regional reference area); ANM = Native Forest (control baseline).

Table S3. Summary of non-parametric comparative tests, parametric validation, and correlation coefficients for total mercury (HgT) concentrations.

**Comparison PEI vs ASH**	Mann–Whitney U $p < 0.001$; Welch t $p < 0.001$
**Correlation canopy × litter**	Pearson $r = 0.91$ ($p = 0.00025$);<br>Spearman $\rho = 0.81$ ($p = 0.008$)
**Enrichment Ratio ($Ratio_C$)**	1.39 – 4.70

The Mann–Whitney U test and Welch's t-test (conducted on log-transformed data) evaluate the significance of differences between the high-exposure urban center (PEI) and the reference area (ASH). Pearson (*r*) and Spearman (*p*) coefficients describe the strength and monotonicity of the paired canopy-litter relationship. The Enrichment Ratio ($Ratio_C$) represents the range of individual calculations defined by $[HgT]_{Litter}/[HgT]_{Canopy\ Leaf}$

Table S4. Geostatistical variogram models and cross-validation statistics for HgT concentrations in canopy leaves across the studied municipalities

Parameter	Peixoto de Azevedo (PEI)	Nova Santa Helena (ASH)
**Variogram Model**	Gaussian	Exponential
**Nugget (C_0)**	23 422.5	0.0
**Structured Sill (C_1)**	18 563.6	1 396.5
**Total Sill ($C_0 + C_1$)**	41 986.1	1 396.5
**Range (α, meters)**	5 275.4	30.41
**Cross-Validation RMSE (μg kg^{-1})**	123.9	35.28
**Cross-Validation ME (μg kg^{-1})**	−12.7	−0.09
**Cross-Validation MSD**	— *(not reported)*	0.93
**Model Diagnostics / Notes**	Exploratory mapping model; uncertainty remains substantial due to localized hotspots and strong small-scale variability.	Model stable; suitable for exploratory kriging interpolation; low bias and well-calibrated prediction variance.

C_0 = Nugget variance (representing microscale heterogeneity and measurement error); C_1 = Structured sill variance; $C_0 + C_1$ = Total sill variance; α = Spatial range (meters); RMSE = Root Mean Square Error; ME = Mean Error; MSD = Mean Standardized Deviation. Models were utilized to map localized atmospheric deposition gradients and point-source emissions
